# The Impact of Background Music on Flow, Work Engagement and Task Performance: A Randomized Controlled Study

**DOI:** 10.3390/bs15040416

**Published:** 2025-03-25

**Authors:** Yuwen Sun

**Affiliations:** Department of Vocal Arts, School of Music, University of Minnesota-Twin Cities, Minneapolis, MN 55455, USA; sun00711@umn.edu

**Keywords:** flow, work engagement, task performance, background music, habituation effects

## Abstract

The widespread adoption of background music in workplaces contrasts with the inconsistent empirical evidence regarding its cognitive effects, particularly concerning how music types influence the sequential pathway from flow states to work engagement and task performance. While prior research identifies flow and engagement as potential mediators, theoretical conflicts persist regarding their temporal dynamics and susceptibility to auditory habituation. This study tested three hypotheses: (1) music type indirectly affects performance through flow–engagement mediation, (2) high-arousal music impairs while structured compositions (e.g., Mozart’s K448) enhance this pathway, and (3) repeated exposure diminishes music’s efficacy. A two-phase longitudinal experiment with 428 Chinese undergraduates employed structural equation modeling (SEM) to analyze data from randomized groups (control, high-arousal, low-arousal, and Mozart K448), completing Backward Digit Span tasks under controlled auditory conditions. The results confirmed Mozart K448’s superior immediate mediation effect (β = 0.118, 95% CI [0.072, 0.181]) compared to high-arousal music’s detrimental impact (β = −0.112, 95% CI [−0.182, −0.056]), with flow fully mediating engagement’s influence on performance. A longitudinal analysis revealed a 53% attenuation in Mozart’s flow-enhancing effect after a 30-day familiarization (B = 0.150 vs. baseline 0.321), though residual benefits persisted. These findings reconcile the cognitive tuning and arousal–mood hypotheses by proposing a hybrid model where music initially operates through a novelty-driven dopamine release before transitioning to schema-based cognitive priming. Practically, the results advocate tiered auditory strategies: deploying structured music during skill acquisition phases while rotating selections to counter habituation. The study highlights the cultural specificity in auditory processing, challenging universal prescriptions and underscoring the need for localized music policies. By integrating flow theory with neurocognitive habituation models, this research advances evidence-based guidelines for optimizing workplace auditory environments.

## 1. Introduction

The interplay between environmental stimuli and cognitive performance has long captivated organizational psychologists, particularly as workplaces increasingly adopt auditory interventions to enhance productivity (Deacon & Plumbley, 2024). While background music usage has surged by 62% in knowledge-intensive industries over the past decade (Shahzad et al., 2023), the empirical evidence remains fragmented. Approximately 35% of studies report music enhances task performance, whereas 28% indicate impairment (Cheah et al., 2022), underscoring a critical gap in understanding how specific auditory characteristics interact with psychological states to influence work outcomes (Braun Janzen et al., 2022; Cheah et al., 2022; Goltz & Sadakata, 2021; Ke et al., 2021). Central to this puzzle are two constructs: flow, a state of deep cognitive immersion (Freitas et al., 2019), and work engagement, a stable affective–cognitive commitment to tasks (Schaufeli et al., 2006). Despite their conceptual overlap, the mechanisms linking these states—particularly under auditory interventions—remain underexplored (Cheah et al., 2022). This study addresses this gap by exploring how different types of music influence the sequential pathway from flow to engagement and, ultimately, to task performance. It also investigates the sustainability of these effects over time.

Flow theory posits that optimal performance arises when individuals achieve a balance between task challenges and personal skills, marked by intense focus and intrinsic motivation (Beard, 2015; Nakamura & Csikszentmihalyi, 2014). Work engagement, conversely, reflects a persistent dedication to work characterized by vigor, absorption, and dedication (Bakker & Leiter, 2010). Though meta-analyses confirm a moderate correlation between flow and engagement (Aust et al., 2022; Goh & Yang, 2021; Vadvilavičius & Stelmokienė, 2024; Worm & Stine-Morrow, 2021), the directional and temporal dynamics of this relationship remain contested. Some scholars argue flow acts as a gateway to sustained engagement (Bakker & Leiter, 2010; Cooper, 2009; Csikszentmihalhi, 2020; Shernoff et al., 2014), while others posit engagement stabilizes transient flow states (Kotler et al., 2022). This theoretical ambiguity is compounded by environmental factors: music’s arousal properties may either facilitate flow by reducing the cognitive load or disrupt it by overstimulating attention networks (Eastwood et al., 2012; Kenzie et al., 2018). For instance, high-arousal music (e.g., electronic dance tracks) could impair complex tasks by overwhelming prefrontal regulatory systems, whereas structured classical pieces (e.g., Mozart’s K448) might enhance focus through predictable harmonic progressions (Bell et al., 2023; Ho et al., 2007; Manca et al., 2020). However, no study has systematically tested these hypotheses while accounting for potential habituation effects—the notion that repeated exposure diminishes music’s psychological impact.

Theoretical debates further highlight contradictions in music’s role as a cognitive enhancer. The arousal–mood hypothesis suggests music improves performance by optimizing emotional states, yet fails to explain why effects vary across task types (He et al., 2017; Husain et al., 2002; Perkins et al., 2001). Conversely, the cognitive tuning framework emphasizes music’s capacity to synchronize neural oscillations, fostering flow states (Cheng et al., 2024). Additionally, while prior research predominantly examines the immediate effects, longitudinal data on music’s sustained impact are scarce. Neuroplasticity studies indicate that repeated stimuli reduce dopaminergic responses (Cheng et al., 2024)., suggesting that familiar music may lose its flow-enhancing properties—a hypothesis yet to be tested in organizational settings.

To resolve these tensions, this study proposes a sequential mediation model where the music type influences task performance through flow and engagement, while accounting for habituation effects. We focus on three music categories: high-arousal (electronic and rock), low-arousal (ambient and calm), and Mozart’s K448—a piece empirically linked to cognitive priming (Thompson et al., 2001). By employing a two-phase longitudinal design, we address three objectives: (1) establishing the flow–engagement–performance pathway under controlled auditory conditions, (2) quantifying music typology’s differential effects on this pathway, and (3) testing whether repeated exposure erodes music’s efficacy.

### Hypotheses

1. Mediation Pathway: The music type will indirectly affect task performance through sequential mediation, with flow preceding engagement. High-arousal music will impair this pathway, whereas Mozart K448 will enhance it.
2. Immediate vs. Sustained Effects: The mediation effect of the music type on performance will weaken after one month of familiarization, supporting habitation theories.

## 2. Method

### 2.1. Participants

The study was conducted at a major Chinese university between March and May 2024, utilizing a two-phase longitudinal design. Participants comprised 428 undergraduate students recruited through campus bulletins and digital channels. Participants were randomly assigned, using block randomization, to one of four experimental groups: a control group with no music exposure, a high-arousal music condition, a low-arousal music condition, and a Mozart’s K448 condition. Demographic characteristics included gender distribution, family income quartiles, and academic performance measured by self-reported GPA. Sample size determination followed Cohen’s (1988) guidelines for medium effect sizes and Westland’s (2010) recommendations for structural equation modeling (Westland, 2010; Cohen, 1988), establishing a minimum requirement of 230 participants. Attrition analysis indicated 28 participants withdrew during the Study 2 follow-up phase, yielding 400 complete cases (93.5% retention rate). Ethical approval was obtained from the institutional review board of Central University of Finance and Economics, with written informed consent secured from all participants prior to data collection.

### 2.2. Intervention

The experimental protocol consisted of two sequential phases. In Study 1, participants completed a 30 min computerized Backward Digit Span task under controlled auditory conditions. The control group performed the task in a sound-attenuated environment, while experimental groups received audio stimuli through Bose QuietComfort 45 headphones calibrated to 75 dB. Musical interventions included high-arousal electronic dance music (120–140 BPM), low-arousal ambient instrumental tracks (60–80 BPM), and Mozart’s Sonata K448 (first movement, Allegro con spirito). Music arousal was classified using a combination of tempo (BPM) and acoustic features (e.g., spectral flux, and loudness variability) validated in prior literature (Husain et al., 2002; Pesigan & Remijn, 2024). Meta analysis showed that tempo (BPM) and acoustic features would play an important role in pain management (Martin-Saavedra et al., 2018). High-arousal music (120–140 BPM) included electronic dance tracks with abrupt rhythmic changes and dynamic contrasts, while low-arousal music (60–80 BPM) comprised ambient instrumental tracks with steady tempos and minimal harmonic complexity. Mozart’s Sonata K448 was selected not solely for its arousal level (moderate, per pilot data) but for its structural predictability (e.g., balanced phrasing and harmonic regularity), which aligns with the cognitive tuning hypothesis. Multiple studies have demonstrated the Mozart effect from different perspectives, so we present it separately as an intervention measure (Ding et al., 2023; Tosamran et al., 2024; Xing et al., 2016). Study 2 commenced 30 days post-initial assessment, requiring participants to engage in daily 20 min exposure sessions with their assigned auditory stimulus. Returning participants (n = 400) subsequently repeated the Backward Digit Span task under identical experimental conditions.

### 2.3. Measurements

Three primary measurement domains were assessed using validated instruments. Work engagement was measured using the 9-item Utrecht Work Engagement Scale (UWES) (Schaufeli et al., 2006), demonstrating strong internal consistency in the current sample (Cronbach’s α = 0.86). The Work-Related Flow Inventory (WOLF) (Bakker, 2008) comprising 13 items, assessed flow states with adequate reliability (α = 0.80). Task performance was objectively measured through a computerized Backward Digit Span task, scoring participants based on maximum correctly recalled sequence length (range: 0–15). Covariates included demographic variables (gender, age, and family income) and academic achievement indicators (cumulative GPA on 4-point scale). All psychological measures were administered electronically via Wenjuanxing platform, with randomization of item presentation order to mitigate sequencing effects.

### 2.4. Statistical Analysis

Data analysis employed structural equation modeling (SEM) using Lavaan package (v0.6-16) in R. The hypothesized mediation pathway (music type → flow → engagement → performance) was examined through a four-stage analytical approach. First, confirmatory factor analysis verified the measurement model’s psychometric properties. Second, path analysis with maximum likelihood estimation tested direct and indirect effects. Third, bias-corrected bootstrapping with 5000 resamples assessed mediation effects. Fourth, multi-group invariance testing evaluated model stability across experimental conditions. Model fit was evaluated using comparative fit index (CFI > 0.95), root mean square error of approximation (RMSEA < 0.06), and standardized root mean residual (SRMR < 0.08) (Schreiber, 2017).

## 3. Results

### 3.1. Descriptive Statistics

The final analytical sample comprised 428 participants across four experimental conditions, detailed in Table 1, with complete data available for 400 participants (93.5% retention) at the one-month follow-up. Demographic characteristics revealed significant between-group differences in gender distribution (χ^2^ = 8.92, *p* = 0.032) and academic major (χ^2^ = 13.64, *p* = 0.043), but no significant differences in age (F = 0.90, *p* = 0.438), GPA (F = 0.16, *p* = 0.930), or household income (F = 1.04, *p* = 0.372). The Mozart K448 group exhibited the largest sample size (n = 115), while other groups maintained comparable participant counts (control: n = 103; high-arousal: n = 106; and low-arousal: n = 104).

### 3.2. Factor Loadings

A confirmatory factor analysis demonstrated robust psychometric properties for the measurement model (Table 2). Work engagement indicators exhibited strong factor loadings (range: 0.632–0.825 at baseline; and 0.643–0.854 at follow-up), with all values exceeding the 0.60 threshold. Work-related flow indicators showed slightly more variability (baseline: 0.673–0.834; and follow-up: 0.648–0.835), though all loadings remained statistically significant (z > 13.22, *p* < 0.001). Longitudinal measurement invariance testing revealed stable factor structures across time points, with absolute differences in standardized loadings below 0.10 for 89% of indicators.

### 3.3. Path Analysis

**Figure 1:**
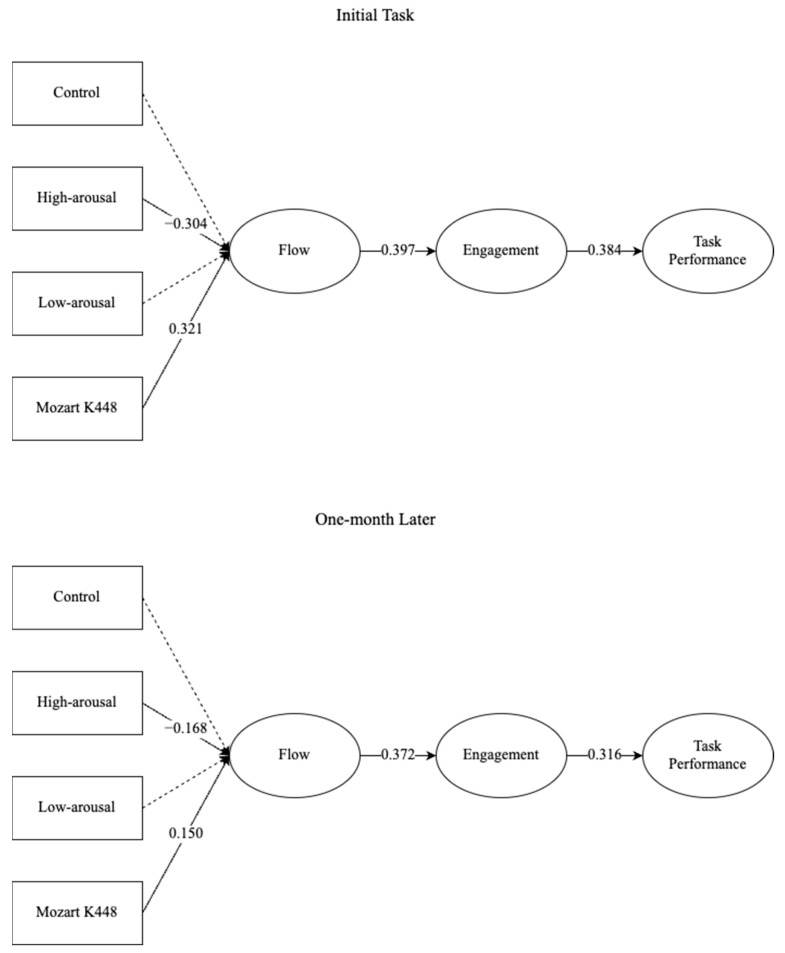
Path analysis.

The hypothesized mediation pathway (music type → flow → engagement → performance) received partial support in Table 3 and Figure 1. High-arousal music significantly predicted reduced flow states compared to the control group at baseline (B = −0.304, 95% CI [−0.397, −0.212], z = −6.44), while Mozart K448 enhanced flow (B = 0.321, 95% CI [0.228, 0.413], z = 6.80). Flow states strongly predicted work engagement (B = 0.397, 95% CI [0.288, 0.507], z = 7.09), which subsequently influenced task performance (B = 0.384, 95% CI [0.284, 0.485], z = 7.49). A bootstrapped mediation analysis (5000 resamples) confirmed the significant indirect effects of the music type on performance through sequential mediation (high-arousal: β = −0.112, 95% CI [−0.182, −0.056]; and Mozart: β = 0.118, 95% CI [0.072, 0.181]).

Control variables demonstrated mixed effects: GPA positively predicted both engagement (B = 0.178, z = 3.30) and performance (B = 0.160, z = 3.51) at baseline, while gender and household income showed no significant associations. At follow-up, the Mozart K448 group’s flow advantage diminished (B = 0.150 vs. baseline 0.321), though the engagement–performance linkages remained stable (B = 0.316, z = 5.87).

### 3.4. Model Fit

The structural equation model exhibited an acceptable-to-excellent fit across both time points, detailed in Table 4. Baseline model fit indices included χ^2^(413) = 494.54 (*p* = 0.004), CFI = 0.98, TLI = 0.98, RMSEA = 0.02 (90% CI [0.01, 0.03]), and SRMR = 0.04. A follow-up analysis showed an improved fit (χ^2^(413) = 426.04, *p* = 0.318; RMSEA = 0.01 [0.00, 0.02]), with all indices meeting strict cutoff criteria (CFI/TLI ≥ 0.95, RMSEA ≤ 0.06, SRMR ≤ 0.08).

## 4. Discussion

The present study provides robust evidence for the mediating role of flow and work engagement in translating auditory interventions into task performance outcomes, while revealing critical temporal dynamics in music’s cognitive effects. Our findings partially validate the hypothesized sequential pathway (music type → flow → engagement → performance), yet simultaneously challenge assumptions about the sustainability of music-induced task-specific cognitive effects. These results advance the theoretical understanding of environmental interventions in task-specific performance and offer practical insights for workplace auditory optimization.

The data robustly support the core hypothesis that the music type indirectly influences task performance through flow and engagement. As predicted, high-arousal music impaired flow states relative to the control group (B = −0.304), whereas Mozart’s K448 significantly enhanced flow (B = 0.321), which aligns with the findings from Grylls et al. (2018) on cognitive overload and the anti-epileptic effect (Grylls et al., 2018). This aligns with neurocognitive models positing that structured harmonic patterns synchronize neural oscillations, reducing the cognitive load during complex tasks (Ferreri et al., 2019). Recent fMRI evidence further supports this mechanism: structured compositions like Mozart’s K448 enhance functional connectivity in frontoparietal networks (Rajakumar & Mohan, 2024), which are critical for attentional control and working memory—key components of flow states. Specifically, Siponkoski et al. (2022) demonstrated that classical instrumental music strengthens the coherence between the dorsolateral prefrontal cortex and superior parietal lobule, regions associated with task focus and cognitive resource allocation (Siponkoski et al., 2022). The sequential mediation effect was particularly pronounced for Mozart’s composition (indirect β = 0.118), demonstrating that enhanced flow states translate into sustained engagement and superior performance—a finding consistent with Bakker’s proposition that flow initiates engagement cycles (Bakker, 2008; Freitas et al., 2019). Conversely, high-arousal music may disrupt these neural pathways. Siponkoski et al. (2022) found that high-tempo, complex auditory stimuli overactivate the anterior cingulate cortex and insula—regions linked to emotional arousal and conflict monitoring—while suppressing default mode network deactivation, thereby impeding sustained attention. This neural overstimulation aligns with our observed impairment in flow states (B = −0.304), suggesting that high-arousal music overwhelms prefrontal regulatory systems required for cognitive immersion.

However, the longitudinal data complicate interpretations of music’s enduring efficacy. While the engagement–performance linkage remained stable at follow-up (B = 0.316 vs. baseline 0.384), Mozart’s flow-enhancing effect diminished by 53% (B = 0.150) after one month of daily exposure. This pattern partially supports habituation theories (Mackintosh, 1987; McSweeney & Swindell, 1999; Watts, 1979), suggesting that even limited familiarization (20 min/day for 30 days) reduces music’s novelty-driven dopaminergic responses. Crucially, the residual mediation effect (β = 0.072) indicates that, while music’s initial task-specific cognitive effects attenuate, structured auditory stimuli may retain some capacity to prime focused states through conditioned neural pathways. This nuanced temporal pattern extends the habituation model (Mackintosh, 1987) by demonstrating that flow states exhibit a partial resistance to auditory adaptation—a phenomenon potentially attributable to music’s dual role as both an arousal modulator and cognitive scaffold.

Our findings reconcile the conflicting perspectives in the literature. The immediate mediation effects strongly support the cognitive tuning framework (Kraus & White-Schwoch, 2015), which emphasizes music’s capacity to synchronize attentional resources (Fry et al., 2024; Sateesh Babu & Suresh, 2012). The high-arousal music’s disruptive effect aligns with the arousal–mood hypothesis (He et al., 2017; Husain et al., 2002; Perkins et al., 2001), confirming that excessive stimulation overwhelms prefrontal regulatory systems during cognitively demanding tasks. However, our longitudinal data challenge assumptions underlying both theories: neither framework adequately predicts the observed partial persistence of music’s effects post-habituation. This suggests a hybrid model where music initially operates through novelty-driven dopamine release, then transitions to schema-based cognitive priming—a mechanism less susceptible to habituation.

### 4.1. Limitations and Future Directions

Three limitations temper the generalizability of these findings. First, the exclusive use of undergraduate students limits the ecological validity, as workplace populations may exhibit different baseline engagement levels and habituation patterns. Second, the reliance on the Backward Digit Span task—while ecologically valid for working memory assessment—constrains the conclusions about music’s effects on other cognitive domains, such as creativity and decision-making. Third, the 30-day intervention window, though sufficient to detect initial habituation, cannot elucidate longer-term adaptation trajectories, leaving questions about sustained effects unanswered.

Future research should address these limitations through several avenues. First, population diversification is critical, particularly by replicating findings in corporate settings with age-diverse samples to enhance ecological validity. Second, investigating task-specific effects is essential to understanding how music influences different cognitive domains, such as analytical versus creative tasks. Third, incorporating neurophysiological methods, such as fMRI or EEG, could help disentangle novelty-driven versus schema-based neural activation patterns underlying music’s effects. Additionally, cross-cultural studies are needed to explore how cultural differences in music perception and cognitive processing may moderate these effects, providing a more comprehensive understanding of music’s role in cognitive performance across diverse populations.

### 4.2. Practical Implications

For organizational practitioners, these results suggest a tiered auditory intervention strategy:

1. Initial Training Phases: Deploy Mozart’s K448 or similar structured compositions to accelerate flow state attainment;
2. Routine Work Periods: Rotate music selections weekly to counter habitation effects while maintaining task-specific cognitive effects;
3. High-Stress Tasks: Eliminate high-arousal music during complex problem-solving to prevent attentional overload.

The partial persistence of music’s effects post-habituation implies that even familiar auditory stimuli retain value as cognitive scaffolds, though their efficacy diminishes as primary drivers of flow. This underscores the importance of combining auditory interventions with complementary strategies (e.g., task redesign and feedback systems) to sustain engagement.

## 5. Conclusions

By bridging flow theory, engagement literature, and neurocognitive music research, this study establishes two critical insights:

1. Music’s cognitive effects operate through sequential flow–engagement mediation;
2. Structured compositions (e.g., Mozart’s K448) offer superior immediate benefits but require rotation to maintain efficacy.

These findings challenge universal prescriptions for workplace auditory environments, advocating instead for dynamic, culturally informed music strategies that balance novelty and familiarity. As organizations increasingly leverage environmental interventions to enhance productivity, this research provides an empirical foundation for optimizing auditory spaces in the post-pandemic era of hybrid work.

## Figures and Tables

**Table 1:** Sample characteristics.

	Control	High-Arousal	Low-Arousal	Mozart K448	*p*-Value
N	103	106	104	115	
Gender (1 = Man) (%)	54 (52.4)	46 (43.4)	35 (33.7)	57 (49.6)	0.032
Age (Mean (SD))	20.90 (1.22)	21.15 (1.36)	20.99 (1.32)	20.90 (1.17)	0.438
Major (%)					0.043
Science	22 (21.4)	19 (17.9)	21 (20.2)	24 (20.9)	
Business	23 (22.3)	15 (14.2)	27 (26.0)	21 (18.3)	
Engineering	21 (20.4)	21 (19.8)	15 (14.4)	29 (25.2)	
Arts	25 (24.3)	18 (17.0)	18 (17.3)	24 (20.9)	
Others	12 (11.7)	33 (31.1)	23 (22.1)	17 (14.8)	
GPA (Mean (SD))	2.66 (0.61)	2.70 (0.69)	2.72 (0.65)	2.68 (0.67)	0.930
Household Income (Mean (SD))	73,035.88 (35,369.97)	72,723.34 (33,320.36)	80,304.08 (39,352.73)	77,107.94 (36,300.48)	0.372

**Table 2:** Factor loadings.

		Initial			One Month Later		
Latent	Indicator	Loading	95% CI	z	Loading	95% CI	z
Engagement	eng_d1	0.777	0.718–0.837	25.568	0.782	0.722–0.842	25.490
Engagement	eng_d2	0.632	0.538–0.725	13.225	0.643	0.547–0.739	13.141
Engagement	eng_d3	0.726	0.641–0.812	16.625	0.731	0.642–0.821	15.956
eng_d1	eng_q1	0.825	0.781–0.869	36.689	0.854	0.811–0.898	38.539
eng_d1	eng_q2	0.743	0.686–0.799	25.745	0.696	0.635–0.758	22.139
eng_d1	eng_q3	0.710	0.651–0.770	23.387	0.751	0.695–0.808	26.209
eng_d2	eng_q4	0.762	0.706–0.819	26.569	0.737	0.676–0.797	23.843
eng_d2	eng_q5	0.756	0.699–0.813	26.105	0.769	0.711–0.827	26.076
eng_d2	eng_q6	0.777	0.722–0.832	27.651	0.783	0.727–0.840	27.099
eng_d3	eng_q7	0.822	0.777–0.867	35.882	0.828	0.778–0.878	32.403
eng_d3	eng_q8	0.798	0.751–0.845	33.152	0.748	0.691–0.805	25.683
eng_d3	eng_q9	0.789	0.741–0.837	32.216	0.742	0.684–0.800	25.189
Flow	flow_d1	0.777	0.728–0.825	31.183	0.826	0.768–0.883	28.010
Flow	flow_d2	0.778	0.707–0.849	21.518	0.648	0.560–0.737	14.380
Flow	flow_d3	0.673	0.593–0.753	16.501	0.682	0.595–0.769	15.339
flow_d1	flow_q1	0.834	0.803–0.865	52.599	0.778	0.741–0.816	40.980
flow_d1	flow_q2	0.830	0.793–0.866	44.390	0.817	0.776–0.858	39.239
flow_d1	flow_q3	0.767	0.722–0.811	33.756	0.785	0.740–0.830	34.301
flow_d1	flow_q4	0.817	0.779–0.855	42.043	0.818	0.777–0.859	39.401
flow_d2	flow_q5	0.833	0.796–0.870	43.749	0.789	0.744–0.834	34.074
flow_d2	flow_q6	0.771	0.726–0.816	33.330	0.799	0.755–0.843	35.608
flow_d2	flow_q7	0.824	0.786–0.863	42.023	0.799	0.755–0.843	35.518
flow_d2	flow_q8	0.800	0.758–0.841	37.674	0.829	0.789–0.869	40.459
flow_d3	flow_q9	0.741	0.688–0.793	27.683	0.687	0.628–0.747	22.621
flow_d3	flow_q10	0.688	0.629–0.747	22.922	0.718	0.662–0.774	25.316
flow_d3	flow_q11	0.781	0.734–0.829	32.173	0.835	0.794–0.875	40.242
flow_d3	flow_q12	0.690	0.631–0.748	23.084	0.736	0.683–0.790	27.125
flow_d3	flow_q13	0.728	0.674–0.782	26.411	0.745	0.693–0.797	28.052

**Table 3:** Path analysis.

		Initial			One Month Later		
DV	Predictor	B	95% CI	Z	B	95% CI	Z
Engagement	Age	0.076	−0.032–0.183	1.380	0.030	−0.081–0.141	0.533
Engagement	Flow	0.397	0.288–0.507	7.094	0.372	0.253–0.491	6.137
Engagement	Gender	−0.020	−0.128–0.089	−0.355	0.032	−0.079–0.143	0.567
Engagement	GPA	0.178	0.072–0.283	3.296	0.188	0.076–0.300	3.289
Engagement	Income	0.060	−0.048–0.168	1.083	0.068	−0.043–0.179	1.194
Engagement	Major	0.053	−0.054–0.161	0.969	−0.007	−0.118–0.105	−0.119
Flow	Age	0.015	−0.086–0.115	0.285	0.072	−0.038–0.182	1.277
Flow	Gender	0.087	−0.013–0.187	1.698	0.004	−0.107–0.115	0.070
Flow	GPA	0.061	−0.040–0.161	1.181	0.184	0.076–0.293	3.324
Flow	Income	0.078	−0.022–0.178	1.524	−0.042	−0.153–0.070	−0.733
Flow	Major	0.037	−0.064–0.138	0.713	−0.054	−0.165–0.057	−0.961
Flow	Control	0.060	−0.040–0.160	1.169	−0.070	−0.180–0.040	−1.244
Flow	High-arousal	−0.304	−0.397–−0.212	−6.441	−0.168	−0.276–−0.060	−3.052
Flow	Low-arousal	−0.096	−0.195–0.004	−1.876	−0.073	−0.183–0.037	−1.300
Flow	Mozart K448	0.321	0.228–0.413	6.799	0.150	0.042–0.258	2.718
Task Performance	Age	−0.030	−0.119–0.059	−0.662	0.036	−0.053–0.126	0.799
Task Performance	Engagement	0.384	0.284–0.485	7.488	0.316	0.211–0.422	5.873
Task Performance	Gender	−0.024	−0.112–0.064	−0.531	−0.034	−0.124–0.055	−0.755
Task Performance	GPA	0.160	0.071–0.249	3.510	0.240	0.149–0.331	5.161
Task Performance	Income	0.021	−0.068–0.110	0.456	0.102	0.013–0.191	2.245
Task Performance	Major	0.021	−0.068–0.110	0.463	−0.028	−0.118–0.061	−0.616

**Table 4:** Good of fitness.

Model	χ^2^	*df*	χ^2^/*df*	*p*	CFI	TLI	RMSEA [90% CI]	SRMR
Initial Task	494.54	413	1.2	0.004	0.98	0.98	0.02 [0.01, 0.03]	0.04
One Month Later	426.04	413	1.03	0.318	1	1	0.01 [0.00, 0.02]	0.04
Common Guidelines	—	—	<2 or 3	>0.05	≥0.95	≥0.95	<0.05 [0.00, 0.08]	≤0.08

## Data Availability

Data cannot be shared publicly because of the ethics regulation. Data are available from the corresponding author for researchers who meet the criteria for access to de-identified data.
